# The importance of biomarker development for monitoring type 1 diabetes progression rate and therapeutic responsiveness

**DOI:** 10.3389/fimmu.2023.1158278

**Published:** 2023-05-15

**Authors:** Maxwell J. Fyvie, Kathleen M. Gillespie

**Affiliations:** Diabetes and Metabolism, Bristol Medical School, University of Bristol, Bristol, United Kingdom

**Keywords:** islet autoantibodies, genetics, exocrine, endocrine, trypsinogen, cfDNA (cell-free DNA), pancreatic enzymes, microRNAs

## Abstract

Type 1 diabetes (T1D) is an autoimmune condition of children and adults in which immune cells target insulin-producing pancreatic β-cells for destruction. This results in a chronic inability to regulate blood glucose levels. The natural history of T1D is well-characterized in childhood. Evidence of two or more autoantibodies to the islet antigens insulin, GAD, IA-2 or ZnT8 in early childhood is associated with high risk of developing T1D in the future. Prediction of risk is less clear in adults and, overall, the factors controlling the progression rate from multiple islet autoantibody positivity to onset of symptoms are not fully understood. An anti-CD3 antibody, teplizumab, was recently shown to delay clinical progression to T1D in high-risk individuals including adults and older children. This represents an important proof of concept for those at risk of future T1D. Given their role in risk assessment, islet autoantibodies might appear to be the most obvious biomarkers to monitor efficacy. However, monitoring islet autoantibodies in clinical trials has shown only limited effects, although antibodies to the most recently identified autoantigen, tetraspanin-7, have not yet been studied in this context. Measurements of beta cell function remain fundamental to assessing efficacy and different models have been proposed, but improved biomarkers are required for both progression studies before onset of diabetes and in therapeutic monitoring. In this mini-review, we consider some established and emerging predictive and prognostic biomarkers, including markers of pancreatic function that could be integrated with metabolic markers to generate improved strategies to measure outcomes of therapeutic intervention.

## 1 Introduction

Type 1 diabetes (T1D) results from autoimmune destruction of insulin-producing pancreatic β-cells (1). The condition has a variable incidence rate of between 3.9-57.4/100,000 depending on the country, and annual incidence rates are increasing at approximately 3-4% worldwide (2). Increased incidence was originally reported in those diagnosed under 5 years of age (3) and this was shown to result from a shift to lower age at onset, and not an overall increased incidence across all age groups (4, 5). However, the Centre for Disease Control report from 2002-2015 shows a sharp increase in incidence of T1D in those diagnosed over age 5, most significantly in black, Hispanic, Asian and Pacific Islander populations (6).

The natural history of T1D is increasingly well understood, particularly in children, making it possible to accurately identify individuals “at risk” of future T1D through islet autoantibody screening. This has facilitated clinical trials to delay the onset of T1D, which recently resulted in the regulatory approval in the USA of teplizumab, an anti-CD3 monoclonal antibody. Teplizumab treatment was shown to provide a delay onset of T1D by >2 years on average in "at risk" individuals (7). There is now increased focus on the optimal strategies to:

1. Identify those at risk; children and adults, both relatives of individuals with T1D and those in the general population for additional clinical trials.
2. Monitor the effectiveness of new therapies.

Biomarkers for a disease can be either predictive, prognostic, or both. In T1D, predictive biomarkers, usually islet autoantibodies, are used to assess risk of clinical diagnosis while prognostic biomarkers, for instance measures of beta cell function, are used to monitor disease progression rate. This mini-review provides a brief “snap-shot” of the current status of prediction and highlights the need for improved prognostic biomarkers.

Crucial to monitoring outcomes of immunomodulatory agents is the phase in the natural history when therapeutic intervention occurs. Primary prevention trials rely on identifying those at genetic risk before the autoimmune process has begun. Current examples are trials within the Global Platform for the Prevention of Autoimmune Diabetes (GPPAD) launched in 2015 (8). GPPAD brings together several centres in Europe where neonates are screened for genetic risk of T1D prior to entry into primary prevention trials including POInT (9) and SINT1A (10). In addition, multiple efforts are ongoing in the USA, Australia, Europe, and the UK to screen for risk of ongoing autoimmunity in children and, more recently, in adults.

**Figure 1:**
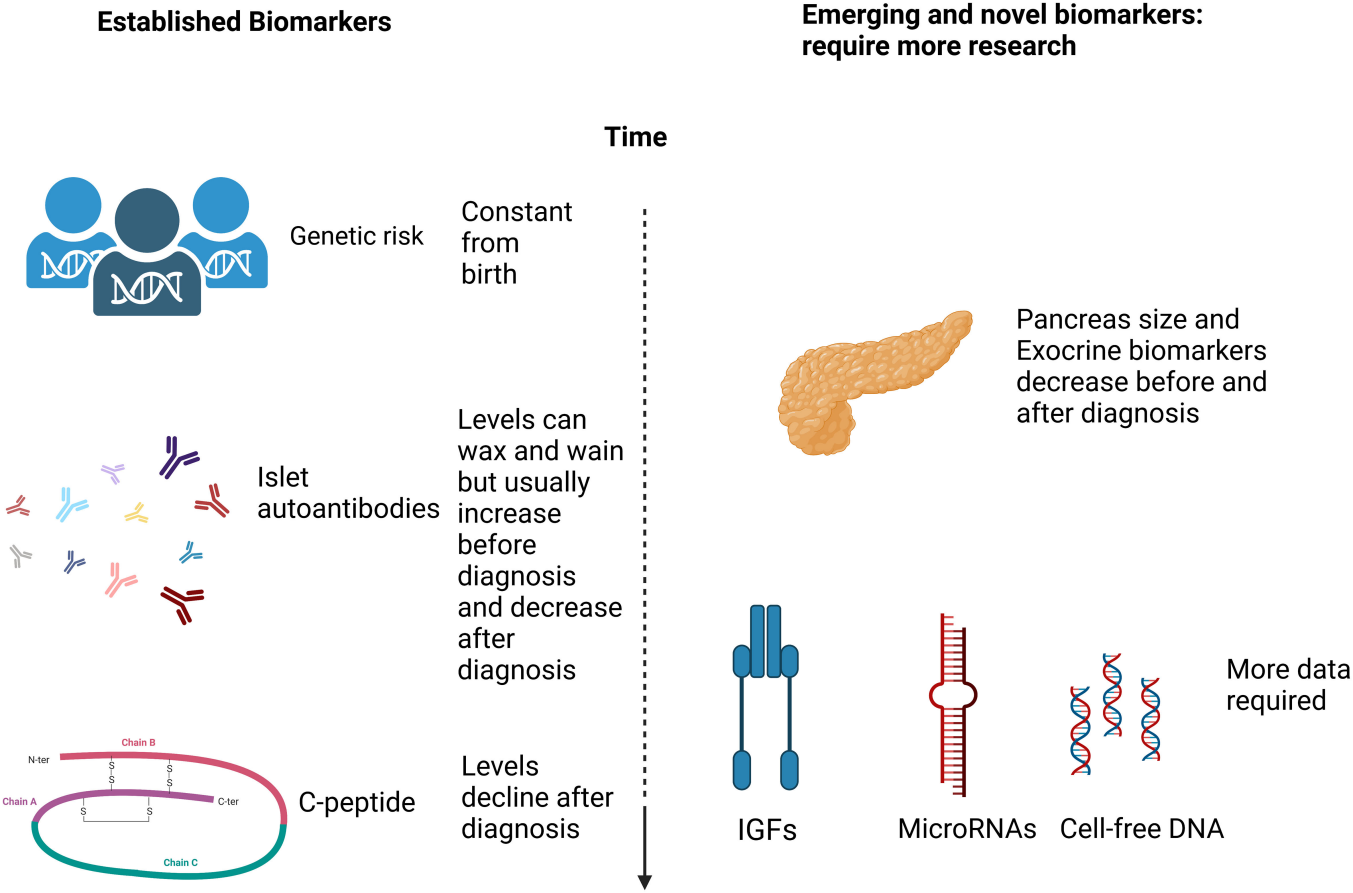
A simplified schematic diagram (created in Biorender.com) of the markers discussed with regard to T1D prediction and prognosis.

Much has been learned about risk calculations and screening approaches from studies of first-degree relatives including BABYDIAB (11); DIPP (12); DAISY (13); TrialNet (14); the Belgian Diabetes Registry (15), the Bart’s Oxford (BOX) study (16) and INNODIA (17). It is not clear, however, whether risk assessment in families where one individual already has been diagnosed with T1D will reflect the general population. Here we examine the strategies used to identify individuals “at risk” of future autoimmune diabetes and consider some of the key established predictive biomarkers and those emerging biomarkers which may, in the future, add to predictive and prognostic models (**Figure 1**).

## 2 Identifying risk of future T1D for clinical trial recruitment

### 2.1 Genetic Risk

The importance of genetics in susceptibility to T1D has long been recognized; studies of monozygotic twins discordant for diabetes demonstrated that approximately half of risk is attributed to genetic factors and half to unidentified environmental factors (18). Human Leukocyte Antigen (HLA) associations were initially described in the 1970s (19, 20). There are three particularly important haplotypes associated with risk of T1D: DRB1*04-DQB1*03:02 (DR4-DQ8), DRB1*03-DQB1*02:01(DR3-DQ2), and DRB1*15-DQB1*06:02 (DR15-DQ6). DR4-DQ8 and DR3-DQ2 are susceptibility haplotypes, whereas DR15-DQ6 is protective (21) including in adult-onset cases (22). The majority of individuals who develop T1D are positive for one or both susceptibility haplotypes (23) and negative for the protective DR15-DQ6 haplotype (24). However, the high-risk combination of DR3/DR4 has been shown to be decreasing over time (25, 26), which suggests an increase in environmental pressure for developing T1D, but the environmental determinants of T1D remain poorly defined.

Genome-wide association studies (GWAS) have identified more than 60 non-HLA variants associated with T1D (27). These include variants in several genes already identified through case control studies [including *INS* (28); *CTLA-4* (29), and *PTPN22* (30, 31)]. Over the last decade there has been a move away from traditional HLA genetic risk assessment to the cheaper, high-throughput strategy of using tagged SNPs to impute HLA risk combining data from HLA and non-HLA variants to generate genetic risk scores (GRS). These scores are proving particularly important in precision medicine approaches to help classify diabetes at diagnosis (32, 33). In terms of identifying risk of future T1D, a GRS has the potential to be used to identify infants in the general population at increased genetic risk of type 1 diabetes through Guthrie spot screening, and the GPAAD platform has paved the way for roll out of this approach (8).

### 2.2 Humoral risk factors: islet autoantibodies

Despite the heterogeneity of T1D, current consensus classifies the prodrome to T1D as having three stages (34), with Stages 1 and 2 being presymptomatic. Stage 1 is defined by the presence of multiple islet autoantibodies in the blood, *without* dysglycemia; Stage 2 by the presence of multiple islet autoantibodies in the blood *with* dysglycemia and Stage 3 represents the onset of symptomatic disease.

The natural history of type 1 diabetes has been studied intensively since the identification of islet autoantibodies with a combined study of three birth cohorts showing that children with two or more islet autoantibodies before the age of 5 years have a >80% risk of developing T1D by the age of 20 (35). The power of islet autoantibodies to predict T1D was first described in the 1970s (36) showing that Islet Cell Antibodies (ICA) could be detected in the blood before the onset of symptoms. This test involves incubating serum on pancreas sections, is operator dependent and lacks specificity. Although still carried out, it has largely been superseded by individual tests for autoantibodies to the four major autoantigens in T1D: insulin (IAA) (37), glutamic acid decarboxylase (GADA) (38), insulinoma-associated protein 2 (IA-2A) (39), zinc transporter 8 (ZnT8A) (40). Tetraspanin-7 (Tspan7A) is a more recently identified autoantigen for T1D (41) although its utility in predicting T1D is not established (42) and initial data suggest that Tspan7A do not provide much added value for T1D prediction (43). More studies are however needed across the age range of T1D to confirm whether or not Tspan7A will be useful as a biomarker for T1D. IAA are often the first islet autoantibody to appear in young children (44), and are more prevalent in this group (10, 45). However, these are often present at lower levels in the blood, which makes them the most difficult islet autoantibody to measure. Autoimmunity to insulin cannot be distinguished from antibodies to exogenous insulin appearing roughly two weeks after the first insulin injection in T1D cases, and therefore samples need to be tested in this window to be useful for diabetes classification or baseline monitoring in trials. Some children develop GADA first (44) while IA-2A and ZnT8A autoantibodies are rarely the first to appear and are usually seen as evidence of epitope spreading of the autoimmune response.

The gold standard islet autoantibody tests are considered to be radiobinding assays (RBAs): liquid phase assays which use a radiolabeled antigen to capture and measure antibodies. They are highly sensitive, and risk data from large international longitudinal research studies such as TEDDY (44) and TrialNet (14) are based on RBAs. However, there are significant cost and safety issues associated with RBA and they are being replaced by other methods including ELISA, LIPS and ADAP (outlined in more detail on **Table 1**). The performance of these assays is measured through testing of blinded samples in islet autoantibody standardization performance (IASP) workshops associated with the Immunology of Diabetes Society (46). Overall, to facilitate general population screening strategies and future clinical trials in both those “at-risk” and in individuals with diabetes, high throughput and cheap sample collection and islet autoantibody tests are required.

**Table 1:** The most commonly used islet autoantibody tests.

Assay type	Strengths	Weaknesses
Radio-binding assays (RBA) - liquid phase assays, use a radiolabelled antigen to capture and measure antibodies. Considered the ‘gold standard’ for islet autoantibody measurement.	Highly sensitive. Longitudinal data from studies such as DIPP, BABYDIAB, BOX, TEDDY, and TrialNet International are based on RBA.	Radioisotopes are expensive and have short shelf-lives due to radioactive decay. Storage and disposal are tightly regulated for safety and environmental reasons. Limited application in clinical settings.
Bridge enzyme-linked immunosorbent assay (ELISA) – solid-phase assay.	Do not rely on radio-labelled antigen tracers. Commercially available. Triplex for GADA, IA-2A and ZnT8A available.	Recombinant protein needs to be manufactured for solid phase adding to costs. Solid phase can obscure antigen epitopes required for autoantibody binding. Often use larger serum volumes.
Electrochemiluminescence (ECL)	Do not rely on radio-labelled antigen tracers. Uses a lower sample volume than ELISAs (15µl per test). Can be multiplexed to simultaneously detect 3-7 autoantibodies, including the four major islet autoantibodies for T1D (IAA, GADA, IA-2A, and ZnT8A).	Serum requires pre-assay acid treatment. Requires specialist equipment. Consumables are expensive.
Luciferase immunoprecipitation systems (LIPS)	Require small serum volumes for testing (usually 2µl for testing in duplicate). One-day duration (versus 2-3 days by RBA). Require minimal specialist equipment. Non-radioactive. Long shelf-life of Nluc-antigens (months versus weeks with radioisotopes). Enhanced adaptability/scalability for large-scale population screening compared to RBAs. Can be multiplexed. Can be used for GADA, IA-2A, ZnT8R, ZnT8W, and IAA.	Placement of Nluc-reporter in antigen sequence may influence antigen conformation and subsequent autoantibody-antigen binding.
Antibody detection by agglutination PCR (ADAP)	Offers increased sensitivity compared to RBA. Low serum volumes required (1-2µl). Can be multiplexed. PCR-based – potential for very high throughput.	Predictive utility is yet to be fully evaluated in at-risk populations.

## 3 Monitoring efficacy in clinical trials

### 3.1 Islet autoantibodies vs. Markers of metabolic function for clinical trial monitoring

Different approaches are currently used to measure β-cell function and provide different readouts about the health of insulin-producing cells (47). The methods most commonly used in research studies to monitor progression rate in individuals with multiple islet autoantibodies are stimulated tests; oral glucose tolerance tests (OGTT); intravenous glucose tolerance tests (IVGTT) and mixed-meal tolerance tests (44). These are carried out by skilled staff, usually in a hospital setting and form the basis for primary outcomes in most T1D clinical trials. Modeling metabolic data to inform progression rates is becoming increasingly sophisticated (48, 49).

While islet autoantibodies are crucial to identify individuals “at-risk” of T1D for trials to prevent or delay the onset of the condition, few data suggest that they represent useful biomarkers to monitor efficacy in the way that models of beta cell function, including C-peptide and immune cell compartments, can be used (48–51). Firstly, some tests use a positive/negative readout for islet autoantibodies and only recently has there been a focus on the potential usefulness of islet autoantibody level in studies of type 1 diabetes (52, 53). However interestingly, in a TrialNet study blocking the CD28/CD80/CD86 costimulatory axis with CTLA4Ig (Abatacept) in individuals with diabetes, participants with a poor response (resistance: measured by modeling rate of decline of C peptide) had *a transient increase* in activated B cell reprogrammed costimulatory ligand gene expression, and *reduced inhibition* of anti-insulin antibodies (54). Similarly, in the Teplizumab trial (7), the absence of ZnT8A identified individuals most likely to respond to the therapy. This shows that autoantibodies at baseline may be predictive of responses to immunotherapy and substantiates the inclusion of islet autoantibodies in monitoring.

### 3.2 Emerging biomarkers

#### 3.2.1 The exocrine pancreas

The pancreas performs both endocrine and exocrine functions. Most biomarker studies have focused on the endocrine compartment, but broader pancreas abnormalities have long been detected in T1D; a reduction in pancreatic size after diagnosis is well-described (55, 56) and pancreas weight is reduced in T1D patients compared with healthy controls (57).

In 2012 using Magnetic Resonance Imaging (MRI), Williams and colleagues showed that the pancreas is already reduced in size by 25% at diagnosis (58). An Australian study in very young “at diagnosis” cases (median 5.5 yrs.) confirmed pancreatic shrinkage in early onset T1D (59). This suggests that pancreatic shrinkage is already ongoing in pre-diabetes. In a study of 85 children participating in the ENDIA study, levels of Fecal Elastase-1, another marker of pancreatic function, were shown to decrease over time in 28 progressors compared to non-progressors (60). A study of TrialNet participants at diagnosis and in those with islet autoantibodies showed that lower levels of circulating P-amylase and lipase (both exocrine enzymes) can be detected before the onset of clinical symptoms in at-risk adult individuals, but not in children (61). Further evidence for the importance of pancreatic enzymes comes from a recent Mendelian Randomisation study to identify circulating proteins influencing type 1 diabetes susceptibility, which showed that increased levels of serum chymotrypsinogen was associated with a decreased risk of T1D (62). Such changes in volume are surprising, since beta cells represent only 2-3% of the pancreas, but reduced pancreas size is thought to reflect loss of the trophic effects of insulin. Recent studies have shown that the exocrine compartment may provide an important source of robust and straightforward biomarkers to monitor effects of therapeutic intervention.

#### 3.2.2 Enzymes of the exocrine pancreas as biomarkers in T1D

Trypsinogen is the proenzyme precursor of trypsin and is stored in the pancreas to be released as required for protein digestion. Immunoreactive trypsinogen (IRT) is a term used to describe the two main isoforms of trypsinogen: the cationic trypsinogen-1, and the anionic trypsingoen-2, both of which are produced by pancreatic acinar cells (63). IRT is released into the circulation in small amounts and can therefore be detected in the blood/plasma. Serum IRT is the most studied indirect test of pancreatic function. It was developed to diagnose chronic pancreatitis (64) and is used to aid diagnosis of exocrine atrophy in T1D (65–67). An IRT test is also currently used worldwide in neonatal screening for cystic fibrosis (68).

There is reduced exocrine pancreatic function in T1D; IRT concentrations have been shown to be significantly reduced in T1D patients compared with healthy matched controls (54). In 2017, Li and colleagues showed that serum trypsinogen levels were significantly reduced in T1D patients compared with controls, and that this was also the case for multiple islet autoantibody positive subjects compared to those with single islet autoantibodies and healthy controls (69). Further studies have built on these findings to demonstrate the potential of trypsinogen as a predictive biomarker for T1D. In 2021, the same team of investigators expanded their studies to trypsinogen, lipase, and amylase in a larger cohort. They showed that trypsinogen and lipase are significantly reduced in subjects with established and recent-onset diabetes, and in individuals with multiple islet autoantibodies compared with single islet autoantibody positive and control subjects (70). In contrast, amylase levels were reduced only in patients with established T1D. They concluded that a combination of serum lipase and trypsinogen levels together provide the most sensitive serological biomarker of BMI-normalised relative pancreas volume (RPV_BMI_), and this could improve disease staging in pre-T1D, although validation in longitudinal samples from “at-risk” individuals is required.

More recently, a proteomics screen of serum from monozygotic twins discordant for T1D unexpectedly identified exocrine proteins as the top five hits compared to co-twins without diabetes (71). Decreased levels were observed for all five proteins and this was subsequently validated for trypsinogen in a large cohort of individuals with T1D where levels were shown to be significantly lower than in healthy control individuals. They also found that trypsinogen levels were lower in recently-diagnosed cases compared with controls across a broad age range, and multiple linear regression in recently-diagnosed participants showed that trypsinogen levels were associated with insulin dose and diabetic ketoacidosis. Age and BMI were important confounders. Trypsinogen levels <15ng/ml were associated with an increased risk of progression in “at-risk” relatives. Together, these results further validate the potential of trypsinogen and possibly other exocrine enzymes as novel and cost-effective biomarkers to monitor efficacy in clinical trials. However, age and BMI need to be incorporated into all models and longitudinal measures will be essential for the outcomes of interventions to be monitored. MRI of the pancreas in combination with measures of exocrine enzymes is also potentially a powerful, if expensive, tool to monitor direct effects of immunomodulation on the pancreas.

#### 3.2.3 MicroRNAs

MicroRNAs (miRNAs) are emerging as a potential area deserving further study in T1D research and may prove to be future biomarkers for the disease (72). miRNAs are small, non-coding RNAs approximately 20 nucleotides long (73) which have been identified in biological samples relevant to T1D, with some studies reporting their role in T1D pathogenesis. They act as gene expression regulators, primarily by inhibiting translation or by causing mRNA degradation, which obstructs protein synthesis at the post-transcriptional stage. miRNAs can be isolated from most biological specimens, and are durable, being protected by microsomes and exosomes, which develop a protective outer shell for the miRNAs. However, miRNA testing could be challenging to implement in a clinical trial setting. This is because sample analyses currently need to be carried out within 8 hours post-collection for an accurate assessment of miRNA species in the plasma, but most multi-centre trials will not be able to deliver samples to central laboratories within this time span.

MiRNA-21 is a specific miRNA that has been shown to disrupt β-cell development in animal models of T1D when overexpressed (74). MiRNA-21 also targets *bcl-2* gene translation, which results in increased β-cell apoptosis during diabetes development (75, 76). Other specific miRNAs that dysregulate pancreatic function include miRNA-29, which impairs glucose-induced insulin secretion when increased in mouse and human pancreatic islets (77). More recently, plasma levels of five miRNAs were shown to be downregulated in diabetic vs. normoglycaemic mice (78). miR-409-3p was also downregulated in immune islet infiltrates of diabetic mice, and its expression correlated with severity of insulitis. Interestingly, CD8+ central memory T cells were enriched in miR-409-3p. Plasma levels of the microRNA gradually decreased during diabetes development in mice and improved with disease remission after anti-CD3 antibody therapy. However, these results do not necessarily suggest that these miRNAs will be similarly relevant to human T1D, because miRNA data are not fully translatable from rodent to human samples. In human plasma samples, miR-409-3p levels were lower in individuals with recently-diagnosed T1D compared with controls, and levels were inversely correlated with HbA1c levels (78). Studies such as these may suggest the potential of microRNAs to monitor therapeutic intervention in T1D, but they have not yet been studied in individuals at risk for the condition, so much work remains to be carried out to fully validate these molecules as tools for prediction or prognosis.

#### 3.2.4 Insulin-like Growth Factors

Insulin-like growth factors (IGFs) promote glucose metabolism. IGF1 and IGF2 have their availability regulated by IGF-binding proteins (IGFBPs). A recent study by Shapiro et al. found that IGF1 and IGF2 levels were significantly lower in islet autoantibody positive compared with islet autoantibody negative relatives of individuals with T1D, and that IGF1 levels decreased over time in subjects with multiple islet autoantibodies and in those who progressed to T1D, in parallel with decreasing β-cell function (79). This study also found that high-affinity IGFBPs remain unchanged in individuals with pre-T1D, which suggests that total IGF levels may reflect bioactivity. These results indicate that IGF dysregulation occurs both before and after T1D diagnosis, and therefore could be a novel biomarker for disease prediction and monitoring the effects of therapy in secondary prevention trials. Importantly, IGFs could act as metabolic biomarkers in that they reflect metabolic dysregulation and therefore could inform T1D staging.

#### 3.2.5 Cell-Free DNA

Cell-free (cf)DNA refers to β-cell-specific, cell-free DNA fragments that are released into the periphery as β-cells are killed by immune cells. These β-cell-specific cfDNA fragments can be measured, should correlate with β-cell death, and could therefore potentially be the most direct biomarker for β-cell death in T1D. Several years ago, multiple studies focused on methylation-specific cfDNA targets, particularly in the insulin gene, to measure β-cell-specific cell death (80–86). However, the methodology fell out of favour when it was reported that an ultrasensitive assay for detection of a β-cell-specific DNA methylation signature failed to observe increases in β-cell-derived cfDNA in a blinded study of 32 autoantibody-positive subjects at risk for type 1 diabetes, 92 individuals with recent-onset type 1 diabetes, and 38 individuals with long-standing disease (87).

In the meantime however, cfDNA increasingly represents an exciting biomarker in cancer studies; in 2021 the National Health Service in the UK launched a research study to examine cfDNA in 140,000 volunteers, aiming to detect 50 types of cancer before symptoms appear. Sample collection systems for cfDNA have improved significantly with collection of plasma samples in dedicated cfDNA tubes which stabilize the cfDNA fragments as the current standard. Therefore, while cfDNA studies in T1D require further optimization, particularly using multiplex approaches, cfDNA has the potential to become a monitoring biomarker of the future. The exquisite specificity of a biomarker capable of directly measuring beta cell death has to be the ambition when monitoring drug efficacy in T1D.

## 4 Conclusions

The outcome of the Teplizumab trial in individuals “at-risk” of future T1D has energized researchers to broaden strategies to identify single- and multiple-islet-autoantibody-positive children and adults in the general population. These strategies would aim to help prevent diagnosis in diabetic ketoacidosis and offer participation in intervention trials and monitoring. Here, we have reflected on some existing and possible future biomarkers to determine efficacy of interventions, enroll and stratify individuals and, hopefully, be used to match the right patient to the right drug at the right time.

## Author contributions

MF and KG designed the review. MF performed literature searches and wrote the manuscript with oversight by KG. KG is responsible for the integrity of the work as a whole. All authors contributed to the article and approved the submitted version.
